# Both Complexity and Location of DNA Damage Contribute to Cellular Senescence Induced by Ionizing Radiation

**DOI:** 10.1371/journal.pone.0155725

**Published:** 2016-05-17

**Authors:** Xurui Zhang, Caiyong Ye, Fang Sun, Wenjun Wei, Burong Hu, Jufang Wang

**Affiliations:** 1 Gansu Key Laboratory of Space Radiobiology & Key Laboratory of Heavy Ion Radiation Biology and Medicine of Chinese Academy of Sciences, Institute of Modern Physics, Chinese Academy of Sciences, Lanzhou, 730000, China; 2 University of Chinese Academy of Sciences, Beijing, 100049, China; University of California Davis, UNITED STATES

## Abstract

Persistent DNA damage is considered as a main cause of cellular senescence induced by ionizing radiation. However, the molecular bases of the DNA damage and their contribution to cellular senescence are not completely clear. In this study, we found that both heavy ions and X-rays induced senescence in human uveal melanoma 92–1 cells. By measuring senescence associated-β-galactosidase and cell proliferation, we identified that heavy ions were more effective at inducing senescence than X-rays. We observed less efficient repair when DNA damage was induced by heavy ions compared with X-rays and most of the irreparable damage was complex of single strand breaks and double strand breaks, while DNA damage induced by X-rays was mostly repaired in 24 hours and the remained damage was preferentially associated with telomeric DNA. Our results suggest that DNA damage induced by heavy ion is often complex and difficult to repair, thus presents as persistent DNA damage and pushes the cell into senescence. In contrast, persistent DNA damage induced by X-rays is preferentially associated with telomeric DNA and the telomere-favored persistent DNA damage contributes to X-rays induced cellular senescence. These findings provide new insight into the understanding of high relative biological effectiveness of heavy ions relevant to cancer therapy and space radiation research.

## Introduction

DNA damage is unavoidable and intrinsic to cells, and often aggravated by genotoxic stresses such as toxic chemicals, ionizing radiation and DNA replication interference. Fortunately, cells can detect their own levels of damage and eventually dismiss the damage for the benefit of the organism by inducing DNA damage response (DDR) [1]. The DDR enables cells to sense and respond to damaged DNA by arresting cell cycle progression and repairing the damage. If the DNA damage remains unrepaired, cells enter into a permanent state known as cellular senescence [2–6]. The cellular senescent signatures, such as inhibition of proliferation, increase of senescence associated-β-galactosidase activity and changed morphology, develop slowly over several days after the initial DNA damage and are maintained by an ongoing DDR.

The DDR pathway is characterized by activation of sensor kinases (ATM/ATR, DNA-PKcs), and induction of checkpoint proteins such as p53, the cyclin dependent kinase (CDK) inhibitor p21 (also known as p21^WAF1/Cip1^) and retinoblastoma protein (RB), which contribute to cell cycle arrest [7]. DDR signaling or repair proteins can assemble rapidly around the damage sites and be detected as DNA damage foci. Two components that are typically used to detect these foci by fluorescence microscopy are the phosphorylated form of the histone variant H2AX (γH2AX), and the adaptor protein p53-binding protein 1 (53BP1). γH2AX foci or 53BP1 foci represent the sites of double strand breaks (DSBs), both can be used as surrogate marker for DSBs [8, 9].

It has been reported that DNA damage induced cellular senescence was associated with persistent DNA damage foci [10]. But the molecular pathways that distinguish transient from persistent DDR foci are unknown. Some studies show that a large fraction of exogenously induced persistent DDR markers are associated with telomeric DNA in cultured cells and mammalian tissues, and these telomere associated persistent DDR are thought to be important in the maintenance of the senescence phenotype [11, 12].

Heavy ions have high relative biological effectiveness (RBE) value because its linear energy transfer (LET) is high and can produce dense ionizing events along the particle track, while the LET of X-rays is low and X-rays only produce sparse ionizing events [13]. Some studies have revealed that high LET radiation induce complex DNA damage, a unique class of DNA lesions that include two or more individual lesions within one or two helical turns of the DNA molecule [14], is more difficult to repair than individual lesions and in some instances is irreparable [9, 15, 16]. However, the capacity of heavy ions to induce cellular senescence has not been thoroughly evaluated and it is also unclear whether complex DNA damage induced by heavy ion irradiation is responsible for cellular senescence. Here we chose X-rays with LET 4 keV/μm, carbon ion beam (^12^C^6+^) with LET 80 keV/μm and iron ion beam (^56^Fe^17+^) with LET 400 keV/μm for irradiation to elucidate these issues. The subsequent DNA damage and cellular senescence were investigated in human uveal melanoma 92-1cells [17].

## Materials and Methods

### Cell culture and irradiation

Human malignant melanoma A375 cells (A375) and human normal embryonic lung fibroblast cell line MRC5 [18] were purchased from the American Type Culture Collection. Human uveal melanoma 92–1 cells (92–1) [17] were stored in our lab. MRC5 cells was cultured in minimal essential medium (Sigma, USA) supplemented with 10% fetal bovine serum (Hyclone, USA), 100 units/mL penicillin and 100 mg/mL streptomycin and maintained in a 5% CO_2_ humidified incubator (Thermo Scientific, NC, USA) at 37°C. A375 cells and 92–1 cells were cultured in RPMI-1640 medium (31800–105, Gibco) complemented with 10% fetal bovine serum (FBS, 1009–141, Gibco), 100 μg/mL streptomycin and 100 units/mL penicillin in a humidified atmosphere with 5% CO_2_. Cells were seeded in 12-well plates (1×10^5^ cells per well), 35 mm culture dishes (2×10^5^ cells per dish) or 60 mm culture dishes (5×10^5^ cells per dish) and incubated for 48 hours to 70% confluence and irradiated at room temperature with X-rays generated by a Faxitron RX650 (Faxitron Bioptics) at a dose rate of 1 Gy/min (100 kVp, 5 mA, no additional filter). Carbon ions (LET 80 KeV/μm) and iron ions (LET 400 KeV/μm) irradiation were performed at the HIRFL (Heavy Ion Research Facility of Lanzhou, Institute of Modern Physics, Lanzhou, China). The dose rate ranged from 0.2 to 0.3 Gy/min.

### Immunofluorescence

For immunostaining, cells were seeded on sterile coverslips at 4 × 10^5^ cells in each 60 mm culture dishes, cultured for 48 hours, then irradiated and incubated (37°C, 5% CO_2_) for indicated time. The irradiated cells were fixed with 4% paraformaldehyde for 10 min at room temperature and in pre-cooling methanol for 20 min at -20°C, permeabilized with 0.5% Triton X-100 in PBS for 10 min. Nonspecific binding sites were blocked with 5% nonfat-dried milk in PBS for 2 hours at room temperature before probing with primary antibodies. Anti-Ki67 rabbit polyclonal (ab15580, Abcam), anti-pATM rabbit polyclonal (ab81292, Abcam) and anti-53BP1 rabbit polyclonal (ab36823, Abcam) antibodies were used. Secondary antibodies (anti-mouse or anti-rabbit, Santa Cruz) conjugated with Alexa Fluor 488/594 were incubated for 1 hours. Images were obtained using a Zeiss LSM 700 Meta laser scanning confocal microscope (Zeiss). For counting foci, we used the spot detection function of the Imaris software. Quantification of foci was done from images of 100–120 cells for each time point from three independent experiments.

### Immuno-FISH

Immuno-FISH was performed as described [19]. Briefly, cells grown on sterile coverslips were fixed with 4% paraformaldehyde and cold methanol, and then incubated with anti-53BP1 (ab36823, Abcam) antibodies for 2 hours at room temperature as described above. After incubated with the secondary antibody, cells were washed with PBS. FISH with a telomere PNA FISH kit (K5326, DAKO) was carried out in accordance with the manufacturers’ instructions. Images were acquired using a Zeiss LSM 700 Meta laser scanning confocal microscope with a 40×1.4-NA Plan-Apochromat oil immersion objective. To avoid bleed-through effects in double/triple-staining experiments, each dye was scanned independently in a multitracking mode.

### SA-β-Gal staining

Human uveal melanoma 92–1 cells (1 × 10^5^) were plated in 35 mm cell culture dishes and incubated for 48 hours before exposure. At each indicated time point after irradiation, cells were stained with the Senescence Associated β-Galactosidase Staining Kit (C0602, Beyotime) following the standard protocol suggested by the manufacturer. Senescent cells were identified under a light microscope.

### EdU incorporation assay

The EdU incorporation assay was carried out using a Cell-Light EdU *in vitro* Imaging Kit (RiboBio, Guangzhou, China) according to the manufacturer’s instructions. In brief, cells were cultured for 2 h in EdU medium after X-ray irradiation, and then fixed with 4% paraformaldehyde for 30 min at room temperature. Cells were stained with Apollo mixture for 30 min after treatment with 0.5% Triton X-100 for 10 min.

### Apoptosis assay

Hoechst 33342/ propidium iodide (PI) staining: Nuclear DNA in treated cells contained in 12-well plates was visualized by staining with the DNA-specific dye Hoechst 33342/PI at a final concentration of 5 mg/ ml and 100 ug/ ml. Cells were observed immediately with filters for blue fluorescence.

### Measurements of ROS

Adherent cells were rinsed twice with serum-free medium and probed with fluorescent dyes (all from Molecular Probes) prepared in serum-free medium. To detect intracellular ROS, cells were stained with 10 μM 2′,7′-dichlorofluorescein diacetate (DCFH-DA) for 20 min at 37°C, then treated with radiation at indicated dose. ROS levels were assessed by fluorescence microscopy.

### Western blotting analysis

Cells in 60 mm culture dishes or 35 mm culture dishes were collected and lysed with RIPA solution (Beyotime). Samples were centrifuged at 10,000 g for 15 min at 4°C, and total protein concentrations determined from supernatants using the BCA protein assay kit (#500–0114, Bio-Rad). Thereafter, samples were resolved with SDS-PAGE and transferred onto polyvinylidene difluoride (PVDF) membrane (GE healthcare). Membranes were blocked for 2 hours in blocking buffer (5% non-fat dry milk in PBS) and incubated with primary antibodies for 2 hours. Next, membranes were washed three times with PBS containing 0.1% Tween-20, incubated with secondary antibody for 1.5 hours at room temperature, and then washed with PBS containing 0.1% Tween-20. Protein bands were visualized using the enhanced chemiluminescence system (WBKLS0100, Millipore) and exposed to X-ray medical film (6535876, Kodak). GAPDH was used as the loading control. The antibodies employed in this study were anti-p53 (ab2433, Abcam), anti-p21 (sc-397, Santa Cruz), anti-pATM (ab81292, Abcam), anti-pRB (8516, Cell Signaling) anti-RB (554136, Becton Dickinson) and anti-GAPDH (sc-25778, Santa Cruz).

### Plasmids and transfection

The GFP-XRCC1 plasmid was constructed from the RFP-XRCC1 plasmid that has been described previously [20, 21]. The plasmid DNA was transfected into cells with Lipofectamine 3000 (Life Technologies) according to manufacturer’s instructions.

### Statistical analysis

The statistical significance (*p* values) in mean values of two-sample comparison was determined with Students’t-test. A value of *p*<0.05 was considered statistically significant (*) and a value of *p*<0.01 was considered extremely significant (**). All experiments were repeated at least three times and the values shown on graphs represent the means ± s.e.m.

## Results

### Cellular senescence is induced by X-rays or heavy ions in 92–1 cells

**Fig 1:**
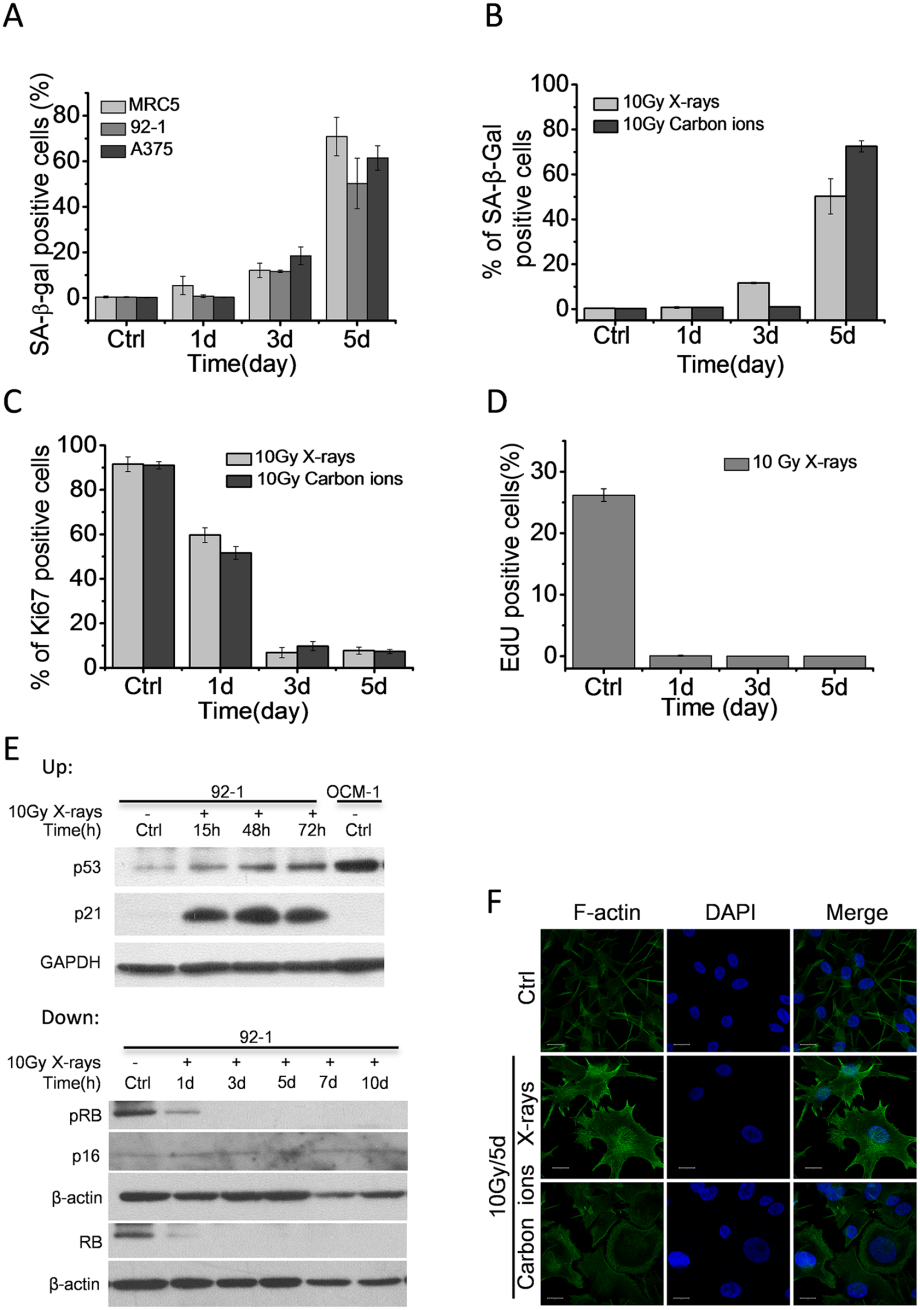
High dose of ionizing radiation induces cellular senescence. (A) Quantification of mean number of SA-β-Gal positive cells in A375, MRC5 and 92–1 cells at various time points post X-ray radiation (10 Gy). (B) Quantification of mean number of SA-β-Gal positive cells in 92–1 cells at various time points post-irradiation. (C) Quantification of mean number of Ki67 positive cells in 92–1 cells at indicated time points post-irradiation. (D) Quantification of mean number of EdU labeled cells in 92–1 cells at indicated time points post X-ray radiation (10 Gy). (E) Up: The protein levels of p53 and p21 expression measured by western blotting, indicating persistent p53/p21 pathway activation after ionizing radiation treatment. OCM-1 was a positive control for p53. Down: The protein levels of p16^INK4a^, pRB and RB expression measured by western blotting. Data are mean ± s.e.m. (n = 3). Ctrl: non-irradiated samples. β-actin: loading control. (F) Representative micrographs of F-actin fluorescence stained 92–1 cells. Scale bar: 20μm. F-actin was stained with phalloidine-FITC.

We first tested whether high dose of ionizing radiation induces cellular senescence in different cell lines. Proliferation-competent human MRC5 fibroblasts, human malignant melanoma A375 cells and human uveal melanoma 92–1 cells were treated with X-ray radiation (10 Gy). We confirmed that these levels of irradiation abolished cell proliferation (data not show) and caused increased activity of senescence-associated β-galactosidase (SA-β-Gal) in these cell lines (Fig 1A), which were consistent with previous studies [2, 3, 11, 12]. However, we also determined that ionizing radiation treatment was unable to induce remarkable cellular senescence in cells with mutant type of p53 (S2 Fig). These results imply that premature senescence is a general response to high dose of ionizing radiation treatment in cells with functional p53/p21 pathway (S1 Fig). To further study, we chose 92–1 cells as cell model.

92–1 cells were exposed to 10 Gy of X-rays or carbon ions. Senescent cells were characterized by expressing high levels of SA-β-Gal and low levels of Ki67 (a cell proliferation marker). The results showed that both carbon ions and X-rays caused a substantial increase in SA-β-Gal positive cells 5 days post-irradiation (Fig 1B), and abolished cell proliferation indicated by Ki67 positive cells 3 days post-irradiation (Fig 1C). As Ki67 staining detected all phases of cells except the rest phase, we also evaluated cell proliferation by EdU assay. EdU pulse labelling only reflected those proliferating cells. As shown in Fig 1D, X-ray radiation (10 Gy) completely abolished cell proliferation of 92–1 cells in 24 hours. In addition, many cells displayed large and flat morphology, a consistent signature of senescence, on the 5th day post-irradiation (Fig 1F). These results suggest the cellular senescence induction by both X-rays and carbon ions.

It has been previously reported that the senescence response crucially depends on the p53/p21 and/or p16^INK4a^/pRB tumor suppressor pathways [22, 23]. Active p53 is required for both establishment and maintenance of senescence in many human cells. And p16^INK4a^ expression is usually upregulated at late stage of senescence. Therefore, we further detected the protein levels of p53, p21, p16^INK4a^, RB and pRB in 92–1 cells exposed to 10 Gy of X-rays. As expected, p53 and p21 were up-regulated and maintained at high levels after irradiation (Fig 1E up). Although RB and pRB (phosphorylated RB) protein levels were dramatically down-regulated, p16^INK4a^ protein levels were hardly detectable in 92–1 cells regardless of treatment with or without X-rays (Fig 1E down). These results imply that the cellular senescence in 92–1 cells is main established through p53/p21 pathway.

### Radiation induced cellular senescence is dose-dependent and LET-associated

**Fig 2:**
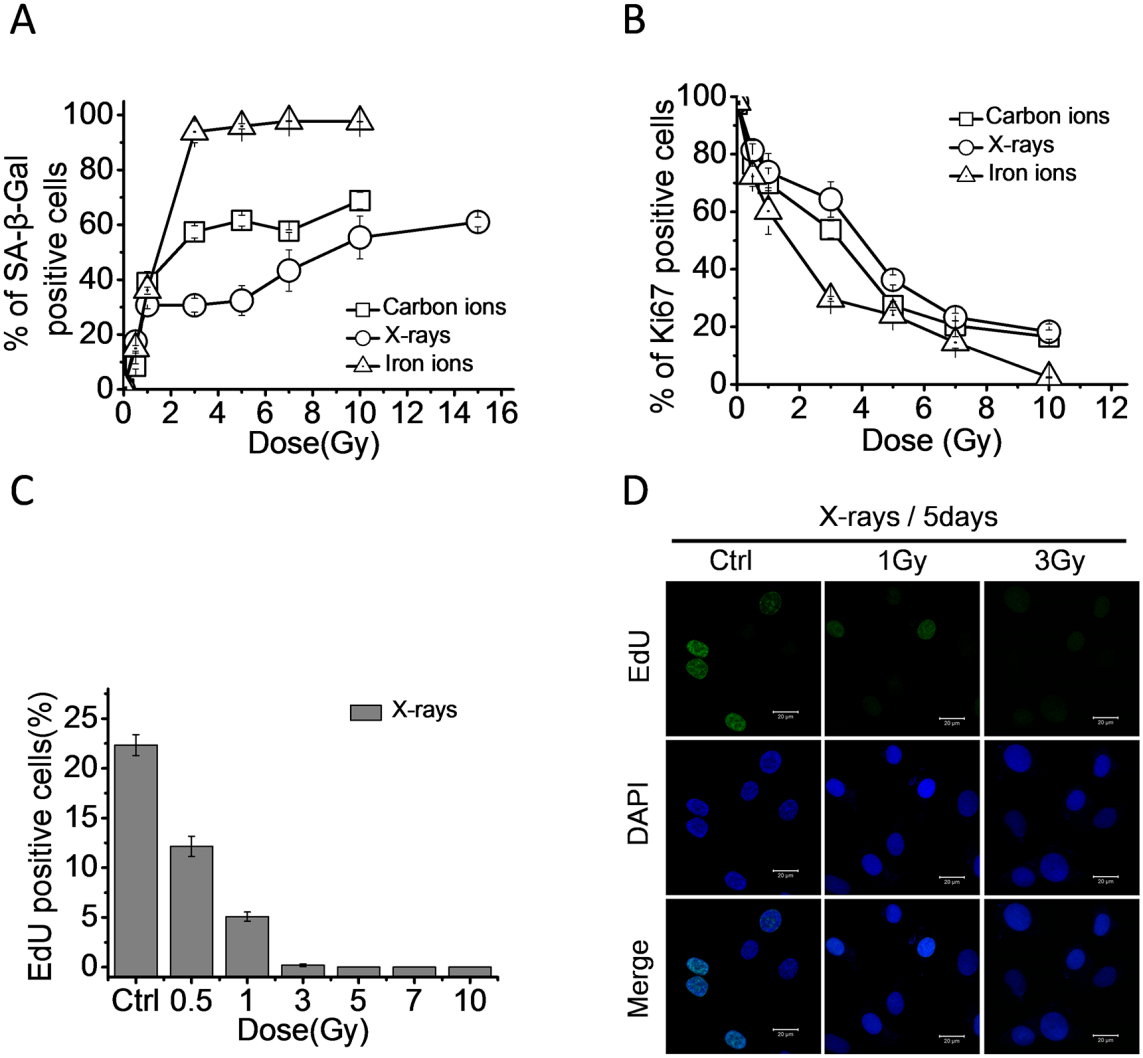
High LET radiation is more efficient to induce cellular senescence than low LET radiation. (A) Quantification of mean number of SA-β-Gal positive cells and (B) Ki67 positive cells in 92–1 cells on the 5th day post treated with 0.5, 1, 3, 5, 7, 10 and 15 Gy of X-rays, carbon ions or iron ions. Data are mean ± s.e.m. (n = 3). (C) Quantification of mean number of EdU positive cells in 92–1 cells on the 5th day post treated with 0.5, 1, 3, 5, 7 and 10 Gy of X-rays. (D) Representative micrographs of EdU labeled 92–1 cells on the 5th day post treated with indicated dose of X-rays. Scale bar: 20 μm.

To explore the relationship between radiation dose and cellular senescence, 92–1 cells were treated with X-rays (LET 4 keV/μm), carbon ions (LET 80 keV/μm) and iron ions (LET 400 keV/μm) at different doses. The senescent cells were detected on the 5th day post-irradiation. As shown in Fig 2A, the SA-β-Gal positive cells remarkably increased with the increasing dose and then saturated at 10 Gy for X-rays, 5 Gy for carbon ions and 3 Gy for iron ions. Consistent with this result, the percentage of Ki67 positive cells decreased with increasing dose (Fig 2B), suggesting the dose-dependency of radiation-induced cellular senescence in 92–1 cells. The result of EdU assay also implied a dose-dependency of radiation-induced cell proliferating arrest in 92–1 cells. The proliferation of 92–1 cells was completely abolished by 3 Gy of X-ray radiation treatments (Fig 2C and 2D).

It was well established that high LET radiation demonstrated high RBE [9]. The high LET specificity of charged particles may contribute to the different capacities of senescence induction. The SA-β-Gal positive cells (Fig 2A) and the Ki67 labeled cells (Fig 2B) showed that carbon ions and iron ions (high LET radiation) were more effective to induce cellular senescence than X-rays at the same dose in 92–1 cells. Taken together, radiation-induced cellular senescence occurs in a dose-dependent manner. Iron ions have the highest LET value and are the most effective radiation to induce cellular senescence among the three types of ionizing radiation (Fig 2A). These results reveal a LET-associated tendency for radiation-induced senescence in 92–1 cells.

### High dose of ionizing radiation induces persistent DDR foci in 92–1 cells

**Fig 3:**
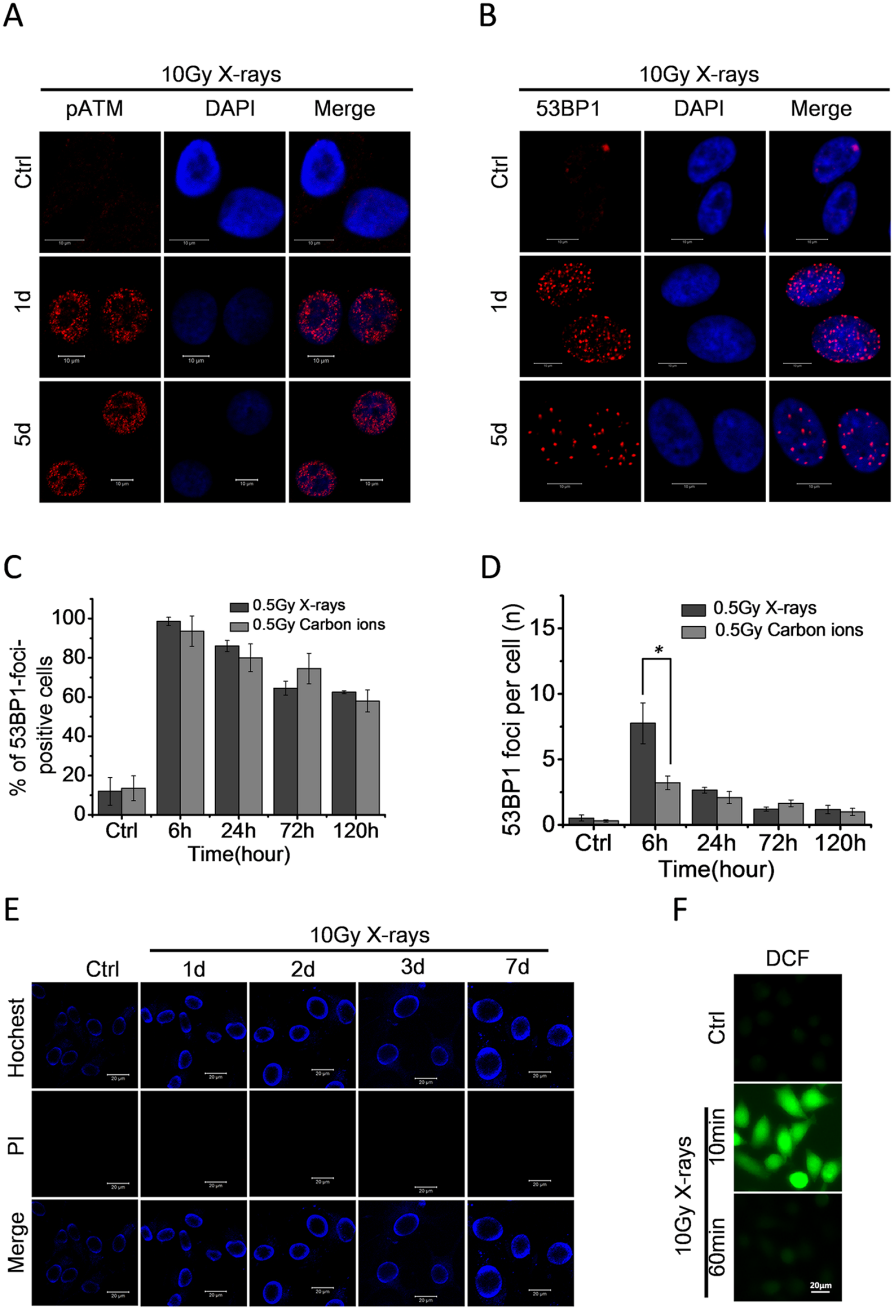
High dose of ionizing radiation induces persistent DDR activation in 92–1 cells. (A, B) Micrographs of DDR foci in 92–1 cells following exposure to 10 Gy of X-rays. Persistent DDR activations are detectable even on the 5th day post-irradiation in the form of pATM foci (A) and 53BP1 foci (B). Scale bar, 10 μm. (C) The fraction of 53BP1foci positive cells (± s.e.m.) and (D) the average number of 53BP1 foci per cell (± s.e.m.) at the indicated time points after irradiation. For the quantification analysis, 100 cells per time point were analyzed (*p<0.05). (E) Micrographs of apoptosis in irradiated 92–1 cells measured by Hochest33342/PI staining; Scale bar, 20mm. (F) Intracellular ROS levels measured by fluorescence microscopy after staining with the fluorescent probe DCF. Scar bar: 20μm.

In order to determine whether radiation induced cellular senescence was caused by persistent DNA damage, we measured the DDR activation after exposure 92–1 cells to 10Gy of X-rays. We observed the formation of pATM (phosphorylated ATM) and 53BP1 foci, which indicated the ATM kinase activation and 53BP1 expression, in 92–1 cells at various time points post-irradiation by immunofluorescence staining. As shown in Fig 3A and 3B, the pATM and 53BP1 foci were activated by high dose of ionizing radiation and kept detectable 5 days post-irradiation.

We also measured the 53BP1 foci in 92–1 cells induced by 0.5Gy of X-rays and carbon ions which could not induce significant senescent phenotypes. The results showed that the majority of damaged DNA was repaired and minimal 53BP1 foci were detectable 5 days post low dose irradiation (Fig 3C and 3D). These results indicate that a sufficient amount of persistent DNA damage caused by high dose of ionizing radiation is essential for the establishment of cellular senescence.

It is well known that radiation not only triggers DNA damage but also apoptosis and oxidative stress/reactive oxygen species (ROS) production in many cell lines. Apoptotic cells demonstrating nuclear condensation and DNA fragmentation can be detected by Hoechst 33342 staining and fluorescence microscopy [24]. To determine whether high dose of radiation treatment induce apoptosis in 92–1 cells, Hoechst 33342/PI staining followed by fluorescence microscopy analysis of apoptosis was performed in 92–1 cells treated with 10 Gy of X-rays. As illustrated in Fig 3E, the radiation treatment did not induce apoptosis in 92–1 cells, which was consistent with our previous studies [2, 3]. We also detected ROS production induced by radiation in 92–1 cells. As shown in Fig 3F, radiation induced a strong increase in intracellular ROS levels within 10 min of cell treatment. However, the DCF fluorescence was decreased to negative control levels during 1 hour after exposure. This implied that the irradiated cells could clear ROS production quickly after radiation exposure.

### High LET radiation is efficient to induce complex DNA damage which is repair-resistant

**Fig 4:**
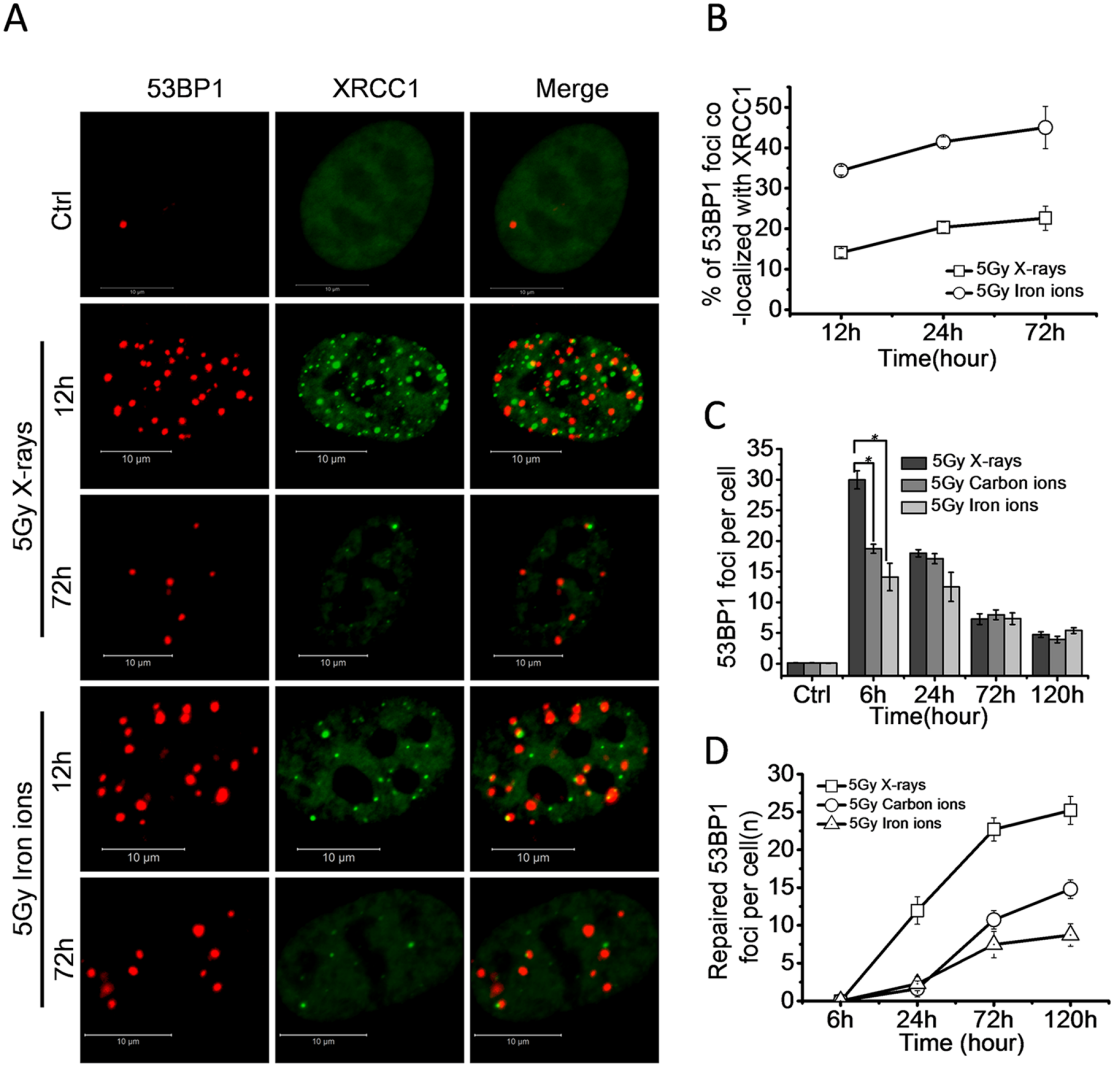
High LET radiation is more efficient to induce repair-resistant complex DNA damage. (A) Representative micrographs showing recruitment and retention of 53BP1 and XRCC1 foci at the sites of damaged DNA induced by 5 Gy X-rays or iron ions in 92–1 cells. Scale bar: 10 μm. (B) Percentage of 53BP1 foci co-localized with XRCC1 foci at indicated times post-irradiation. Data are mean ± s.e.m. (n = 3). For the quantifications analysis, 50 cells were analyzed per time point. (C) The average number of 53BP1 foci per cell (± s.e.m.) at the indicated times post-irradiation. For the quantification analysis, 100 cells per time point were analyzed. (D) The number of repaired 53BP1 foci per cell at indicated times post-irradiation (the number of 53BP1 foci at 6 hours is defined as initial DNA damage). (*p<0.05).

Previous evidence suggests that high LET radiation induces complex DNA damage, which includes two or more individual lesions within one or two helical turns of the DNA [25]. These lesions are more difficult to repair than individual lesions and in some instances are irreparable [9]. To understand the nature of irreparable DNA damage induced by high LET radiation, cells expressing EGFP-XRCC1 were irradiated with 5 Gy of X-rays or iron ions (LET 400 keV/μm). We tested the single strand breaks (SSBs) and double strand breaks (DSBs) by fluorescence tagged XRCC1 foci and immunofluorescence stained of 53BP1 foci, respectively. Examination of high-resolution images of XRCC1 and 53BP1 foci revealed that both X-rays and iron ions induced SSBs and DSBs (Fig 4A). The 53BP1 foci co-localized with XRCC1 foci were complexes of SSBs and DSBs. By using 3D imaging technology, we quantified the number of co-localized foci. As shown in Fig 4B, the percentage of 53BP1 foci co-localized with XRCC1 foci in the cells exposed to iron ions was significantly higher than the percentage of co-localization in cells exposed to X-rays from 12 to 72 hours post-irradiation. At 12 hours, about 34% of 53BP1 foci co-localized with XRCC1 in the cells exposed to iron ions, meanwhile, only 14% of 53BP1 co-localized with XRCC1 in X-ray irradiated cells, indicating that high LET radiation was more efficient to induce complex DNA damage than low LET radiation.

To identify the repair ability of DNA damage induced by different types of radiation, we exposed proliferating 92-1cells to 5 Gy X-ray, carbon ion or iron ion radiation. From the repair kinetics results of 53BP1 foci, it was observed that although the number of 53BP1 foci induced by X-rays was higher at the early time point (6h), the number of 53BP1 foci reduced to the similar levels as that induced by carbon or iron ion radiation at 24 hours post-irradiation (Fig 4C). It was also found that significant number of 53BP1 foci persisted in cells even 5 days after radiation. These results imply that DNA damage induced by high LET radiation is more difficult to repair than damage from low LET radiation (Fig 4D).

Collectively, these results demonstrate that high LET radiation is responsible for the complexity of DNA damage, which is difficult to repair and contributes to the persistency of DNA damage.

### Low LET radiation induced persistent DDR foci are preferentially associated with telomeric DNA

**Fig 5:**
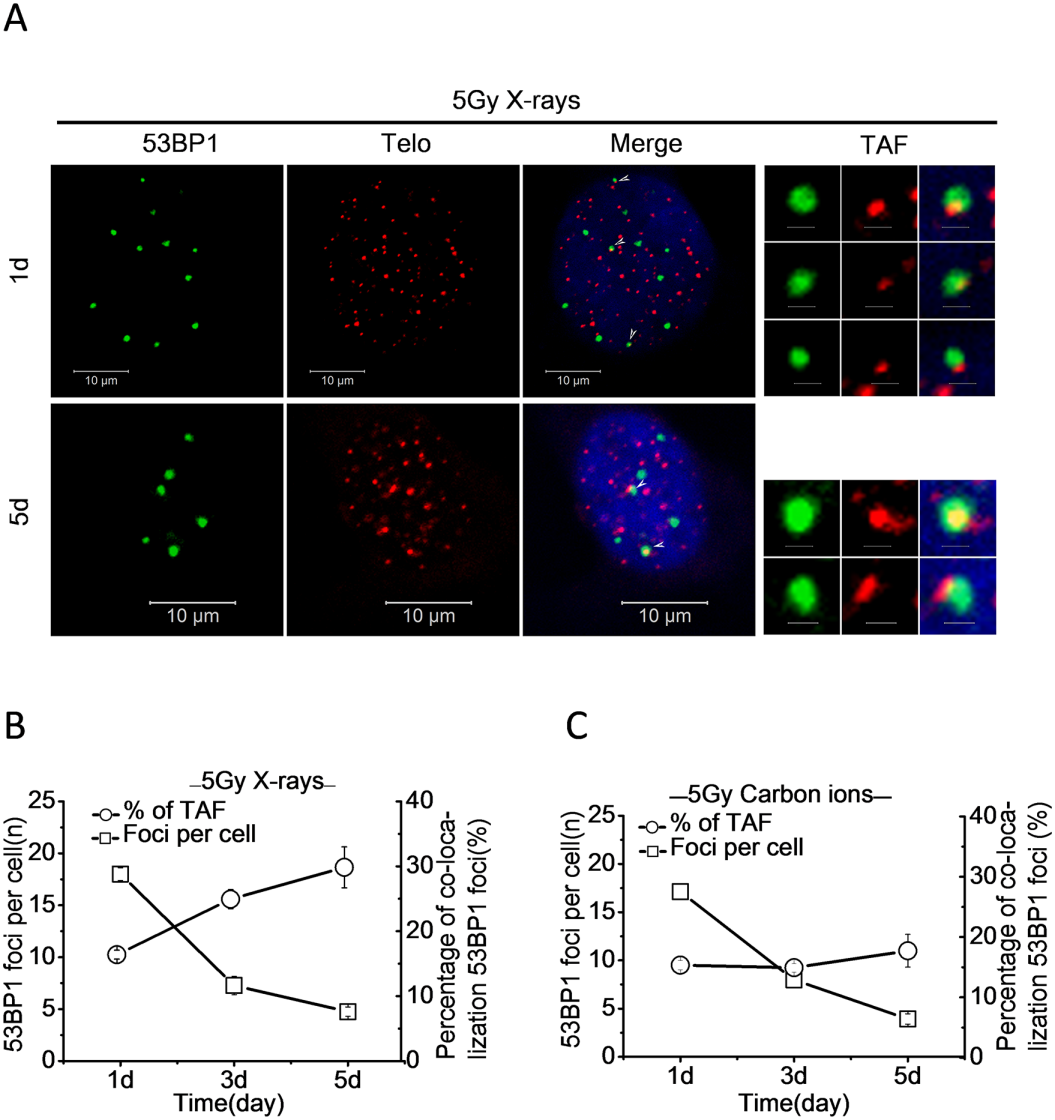
Low LET radiation induced persistent DDR foci are preferentially associated with telomeric DNA. (A) Representative micrographs of co-localizations between 53BP1 foci and telomeric-PNA probes (Telo) in 92–1 cells, at the indicated time points after 5 Gy of X-ray exposure. Scale bar: 10 μm (1μm in TAF panels. TAF: Telomere-associated foci). The average number of 53BP1 foci per cell and the percentage of 53BP1 foci co-localized with telomeres in 92–1 cells exposed to 5 Gy of X-rays (B) or carbon ions (C). Data are mean ± s.e.m. (n = 3). For the quantification analysis, 120 cells were analyzed for 53BP1 foci experiments and 100 cells for co-localization experiments.

Although ionizing radiation was expected to generate DNA damage randomly, the distribution of DDR foci may be uneven due to the specificity of radiation type and different repair ability of DNA damage. It was reported that exogenously induced persistent DDR foci co-localized with telomeres and contributed to cellular senescence. [11, 12] To further illustrate this phenomenon, we checked the distribution of persistent DDR foci on chromatin by immunofluorescence staining of 53BP1 in conjunction with fluorescence *in situ* hybridization (FISH) of telomeric Cy3-conjugated peptide-nucleic acid (PNA) probe (immunoFISH). After treatment of the 92–1 cells with 5 Gy of X-rays or carbon ions, 53BP1 foci and telomere-associated foci (TAF) were detected by unbiased co-localization imaging software at different times (Fig 5A). The results revealed that the average number of 53BP1 foci per cell progressively declined for both X-ray and carbon ion irradiated 92–1 cells (Fig 5B and 5C). With the repairing of DNA damage generated, the percentage of 53BP1 foci co-localized with telomeres continually increased and reached to 30% for X-rays at the 5th day after treatment (Fig 5B). However, the percentage of 53BP1 foci co-localized with telomeres remained steadily around 15% for carbon ions after treatment (Fig 5C). These results suggest that persistent DDR foci are preferentially associated with telomeric DNA and the telomere-favored persistent DNA damage contribute more to cellular senescence caused by low LET radiation.

## Discussion

Substantial evidence demonstrates that X-rays and γ-rays can induce cellular senescence [2, 4, 12]. Recently, heavy ion beams have become of growing interest for application in tumor therapy due to the advantageous physical and radiobiological properties. On the other hand, the proportion of heavy ions in space radiation is low (around 1% of the cosmic rays), but the detrimental effects of heavy ions cannot be neglected due to their high RBE. Compared to X-rays, heavy ions are more effective in killing cells. Up to now, only little study focuses on how heavy ions impact cellular senescence. Here, we demonstrated that heavy ions could induce senescence in 92–1 cells, though heavy ions were charged high atomic number particles while X-rays were uncharged photons. As shown in Fig 2A and 2B, heavy ions with high LET value were more efficient to induce cellular senescence than X-rays at the same dose. In addition, functional activation of p53/p21 pathway in response to stress was crucial for senescent establishment in irradiated cells. Our studies confirmed that p21 is a major regulator for cells to respond to ionizing radiation treatment.

Cellular senescence is a stable condition in which cells are unable to further divide. It was a crucial barrier to suppress cancer development. Our results and other studies confirmed that ionizing radiation, including X-rays, γ-rays and heavy ion beams, could induce cellular senescence in normal and cancer cells [2,26,27]. These results implied that irradiation for radiotherapy of cancers may induce cellular senescence in the tumor cells. This could efficiently arrest cancer cell proliferation by triggering senescence. However, senescent cells also secrete molecules that can stimulate premalignant cells to proliferate and form tumors, suggesting the senescence response is antagonistically pleiotropic [28, 29].

It is well known that radiation not only triggers DNA damage but also ROS production. Many studies show that ROS can induce cellular senescence. Indeed, hydrogen peroxide (H_2_O_2_) is a potent inducer of cellular senescence in many cell types. While exogenous treatment with H_2_O_2_ can promote cellular senescence, endogenous ROS (such as superoxides and hydroxyl radicals) is also implicated in the establishment and maintenance of the irreversible growth arrest. In our study, high dose of radiation treatment triggers ROS production increase quickly, it may active DNA damage response by the activation of p53/p21 pathway and thus contributes to cellular senescence establishment. However, the ROS production declines quickly after irradiation in 92–1 cells. Hence, the mechanisms involved in linking ROS and cellular senescence in our cell model still need to be further studied.

Increasing evidences suggest that chromatin organization regulates the cell’s ability to repair DNA damage [30–33]. Two independent studies confirm that genomes are not uniformly reparable and telomere regions resist DNA damage repair despite a global cellular competence for DNA repair [11, 12]. So exogenous stimuli induced persistent DDR markers are associated with telomeric DNA. However, Asaithamby *et al* suggest that difficulties associated with persistent DNA damage repair are not due to their physical location within the substructure of chromatin, but the complexity of these damaged DNA in nature [9]. We identified that about 34–44% of 53BP1 foci (marker of DSBs) were co-localized with XRCC1 foci (marker of SSBs) in 92–1 cells exposed to iron ions, while the fraction of 53BP1 foci co-localized with XRCC1 foci was only around 14–22% in the cells exposed to X-rays (Fig 4A and 4B), indicating that heavy ions were more efficient to induce complex DNA damage than X-rays. Therefore, we suggest that most of heavy ion induced DNA lesions are complexes of SSBs and DSBs. These lesions are very close to each other, and thus result in the high complexity of DNA damage. Meanwhile, most of X-rays caused DNA lesions are individual SSBs or DSBs, and further apart to each other. In addition, parallel experiments compared the repair kinetics of DNA damage in cells treated with X-rays, carbon ions or iron ions at the same dose. The results showed that most of DNA damage induced by high LET heavy ions was refractory to repair (Fig 4C and 4D), and the irreparable DNA damage presented as persistent DNA damage and triggered the cellular senescence.

On the other hand, a large fraction of repair-resistant DNA damage induced by X-rays located at telomeres. The percentage of telomere-associated persistent 53BP1 foci reached to 30% of the whole genome 53BP1 foci on the 5th day post-radiation (Fig 5B), which was consistent with previous studies [11, 12]. However, the percentage of telomere-associated persistent 53BP1 foci was not increased over time and maintained around 15% (Fig 5C) after carbon ion exposure, suggesting that the complex DNA damage resistant to repair is not due to its physical location within the substructure of chromatin, but the high complexity of DNA damage caused by heavy ions.

**Fig 6:**
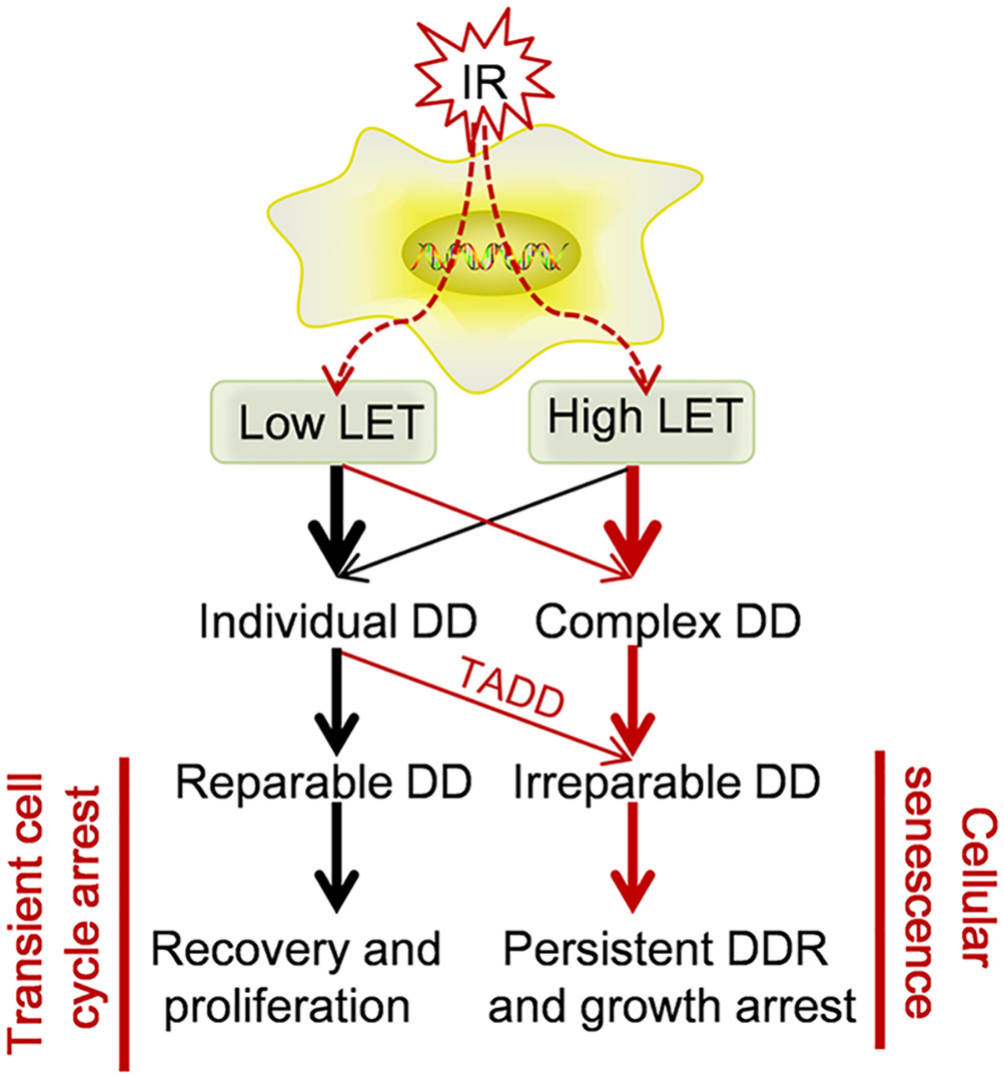
A proposed cellular model with two different outcomes caused by low LET radiation or high LET radiation. DD: DNA damage. TADD: Telomere-associated DNA damage.

On the basis of obtained data, we conclude a cellular model with two different outcomes caused by low LET radiation or high LET radiation (Fig 6). After exposure 92–1 cells to ionizing radiation, the amount and complexity of DNA damage are determined by the dose and specificity of radiation. A sufficient amount of persistent DNA damage is essential for the establishment and maintenance of radiation-induced senescence. Both the complexity and location of DNA damage impact their repair efficiency. The complex DNA damage induced by high LET radiation is difficult to repair, thus presents as persistent DNA damage and is responsible for cellular senescence. In contrast, much more of low LET radiation induced DNA damage are efficient repair and the remained persistent DNA damage is preferentially associated with telomeric DNA. This telomere-favored persistent DNA damage contributes more to the low LET radiation induced cellular senescence.

## Supporting Information

S1 Fig Irradiation induced p53 protein accumulation and p21 expression in *p53*^*+/+*^ cell lines. 92–1, A375 and MCF7 cell lines with wild type of p53 were treated with 10 Gy of X-rays, the protein levels of p53 and p21 expression measured by western blotting, indicating persistent p53/p21 pathway activation after ionizing radiation treatment. Western blots represent three independent experiments.
(TIF)

S2 Fig Irradiation treatment is unable to induce cellular senescence in cell lines with mutant type of p53. 786-O,MGC-803,Hela and OCM-1 cell lines with mutant type of p53 were treated with 10 Gy of X-rays. (A) Quantification of mean number of SA-β-Gal positive cells in these cell lines at various time points post-irradiation. The protein levels of p53 and p21 expression were measured in irradiated MGC-803(B), 786-O(C),Hela(D), OCM-1(E) cells by western blotting assay at various time points post-irradiation.92-1 cells were used as positive control for p21 protein in western blotting assay.
(TIF)

S3 Fig Ionizing radiation induces cellular senescence in a dose-dependent manner in A375 cells. Quantification of mean number of SA-β-Gal positive cells in A375 cells on the 5th day post treated with 0.5, 1, 3, 5, 7 and 10 Gy of X-rays. Data are mean ± s.e.m. (n = 3).
(TIF)

S4 Fig 53BP1 foci mark the sites of DSBs. Representative images showing colocalization of 53BP1 with γH2AX at the sites of DNA damages induced by low-LET X-rays. 92–1 cells and MRC5 cells were exposed to X-rays (5 Gy), fixed after 24h, and immunostained with antibodies against 53BP1 and γH2AX. Images were acquired by using confocal microscopy.
(TIF)
