# Supplementary figures and images for: Both Complexity and Location of DNA Damage Contribute to Cellular Senescence Induced by Ionizing Radiation
Source: PLoS One. 2016 May 17;11(5):e0155725. doi: 10.1371/journal.pone.0155725 (PMC4871470; doi:10.1371/journal.pone.0155725)

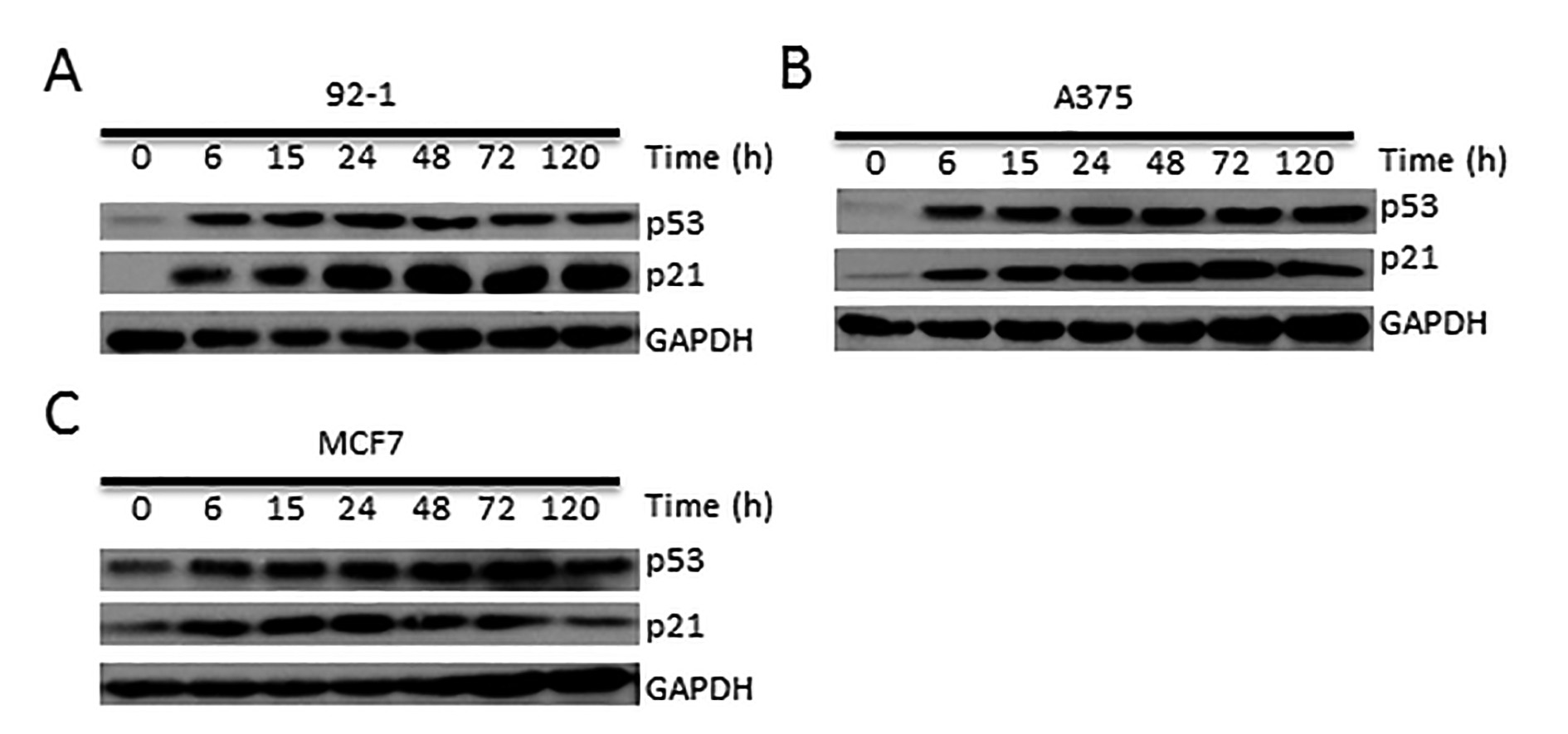

Supplement: S1 Fig — 92–1, A375 and MCF7 cell lines with wild type of p53 were treated with 10 Gy of X-rays, the protein levels of p53 and p21 expression measured by western blotting, indicating persistent p53/p21 pathway activation after ionizing radiation treatment. Western blots represent three independent experiments. (TIF) [file pone.0155725.s001.tif]

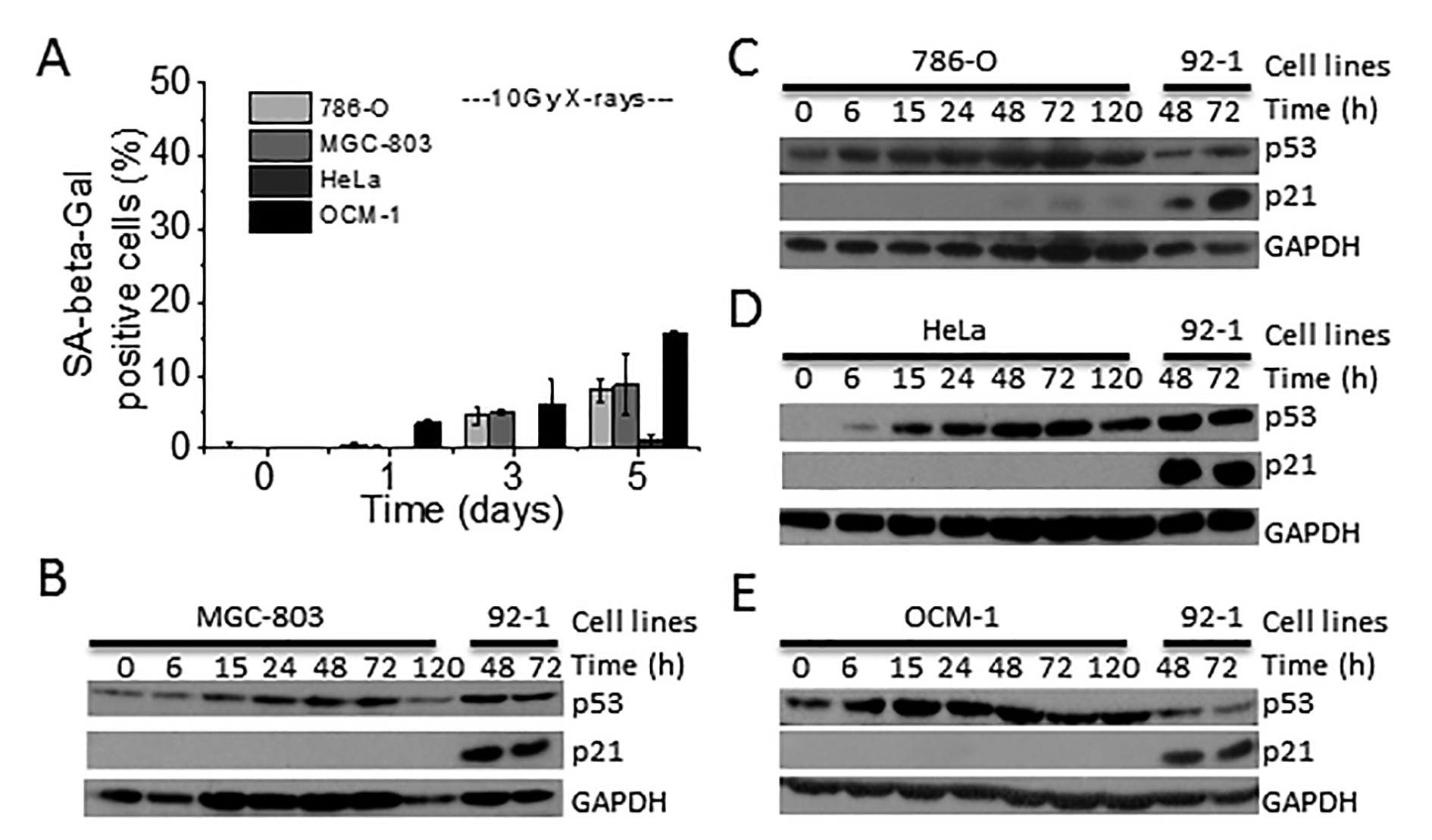

Supplement: S2 Fig — 786-O,MGC-803,Hela and OCM-1 cell lines with mutant type of p53 were treated with 10 Gy of X-rays. (A) Quantification of mean number of SA-β-Gal positive cells in these cell lines at various time points post-irradiation. The protein levels of p53 and p21 expression were measured in irradiated MGC-803(B), 786-O(C),Hela(D), OCM-1(E) cells by western blotting assay at various time points post-irradiation.92-1 cells were used as positive control for p21 protein in western blotting assay. (TIF) [file pone.0155725.s002.tif]

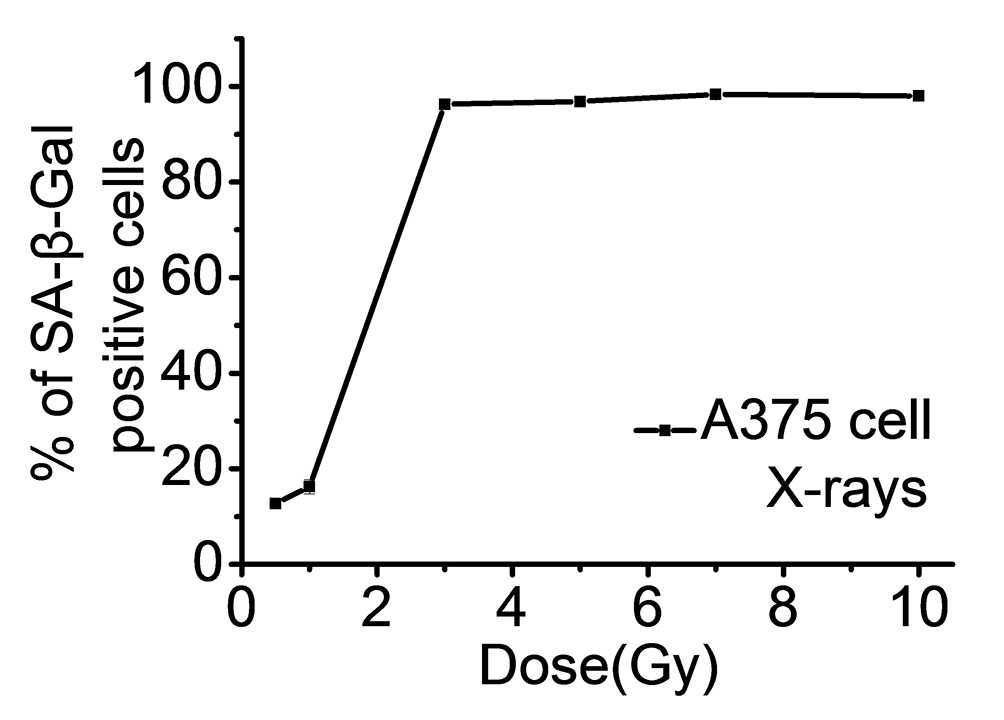

Supplement: S3 Fig — Quantification of mean number of SA-β-Gal positive cells in A375 cells on the 5th day post treated with 0.5, 1, 3, 5, 7 and 10 Gy of X-rays. Data are mean ± s.e.m. (n = 3). (TIF) [file pone.0155725.s003.tif]

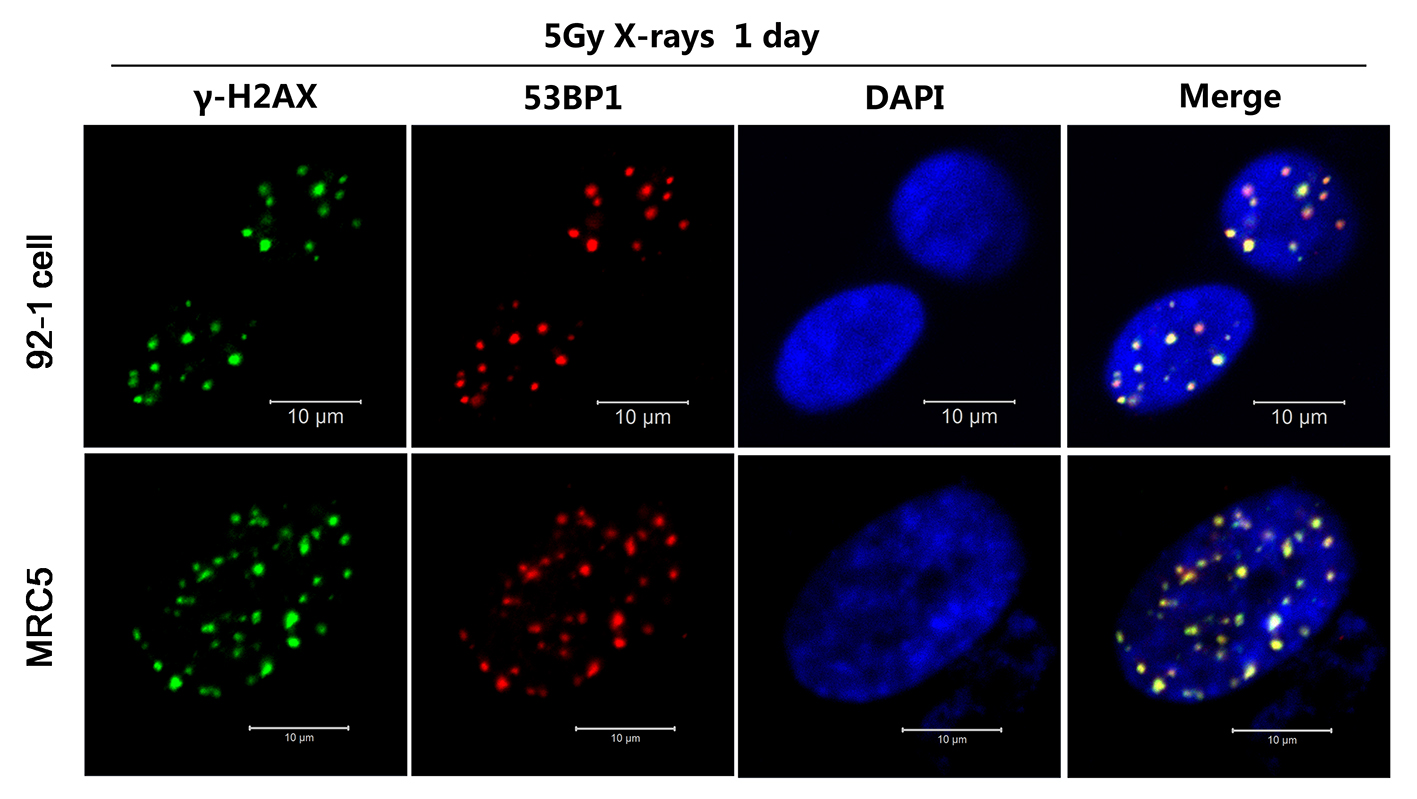

Supplement: S4 Fig — Representative images showing colocalization of 53BP1 with γH2AX at the sites of DNA damages induced by low-LET X-rays. 92–1 cells and MRC5 cells were exposed to X-rays (5 Gy), fixed after 24h, and immunostained with antibodies against 53BP1 and γH2AX. Images were acquired by using confocal microscopy. (TIF) [file pone.0155725.s004.tif]
